# Remodeling of the heart in hypertrophy in animal models with myosin essential light chain mutations

**DOI:** 10.3389/fphys.2014.00353

**Published:** 2014-09-22

**Authors:** Katarzyna Kazmierczak, Chen-Ching Yuan, Jingsheng Liang, Wenrui Huang, Ana I. Rojas, Danuta Szczesna-Cordary

**Affiliations:** Department of Molecular and Cellular Pharmacology, University of Miami Miller School of MedicineMiami, FL, USA

**Keywords:** hypertrophic cardiomyopathy (HCM), transgenic mice, myosin essential light chain, mutation, cardiac remodeling, histopathology

## Abstract

Cardiac hypertrophy represents one of the most important cardiovascular problems yet the mechanisms responsible for hypertrophic remodeling of the heart are poorly understood. In this report we aimed to explore the molecular pathways leading to two different phenotypes of cardiac hypertrophy in transgenic mice carrying mutations in the human ventricular myosin essential light chain (ELC). Mutation-induced alterations in the heart structure and function were studied in two transgenic (Tg) mouse models carrying the A57G (alanine to glycine) substitution or lacking the N-terminal 43 amino acid residues (Δ43) from the ELC sequence. The first model represents an HCM disease as the A57G mutation was shown to cause malignant HCM outcomes in humans. The second mouse model is lacking the region of the ELC that was shown to be important for a direct interaction between the ELC and actin during muscle contraction. Our earlier studies demonstrated that >7 month old Tg-Δ43 mice developed substantial cardiac hypertrophy with no signs of histopathology or fibrosis. Tg mice did not show abnormal cardiac function compared to Tg-WT expressing the full length human ventricular ELC. Previously reported pathological morphology in Tg-A57G mice included extensive disorganization of myocytes and interstitial fibrosis with no abnormal increase in heart mass observed in >6 month-old animals. In this report we show that strenuous exercise can trigger hypertrophy and pathologic cardiac remodeling in Tg-A57G mice as early as 3 months of age. In contrast, no exercise-induced changes were noted for Tg-Δ43 hearts and the mice maintained a non-pathological cardiac phenotype. Based on our results, we suggest that exercise-elicited heart remodeling in Tg-A57G mice follows the pathological pathway leading to HCM, while it induces no abnormal response in Tg-Δ43 mice.

## Introduction

In response to various types of stimuli (genetic, mechanical, hemodynamic, hormonal, physiological, environmental factors or their combination), the heart has the ability to adapt to increased workloads through the hypertrophy of muscle cells (Hunter and Chien, 1999; Lorell and Carabello, 2000). The hypertrophic response is considered physiological if the heart fully adapts to the new loading condition, reaching a new steady state. This type of hypertrophy is characterized by a normal organization of cardiac structure (normal cardiac morphology), normal or enhanced cardiac function and a relatively normal pattern of gene expression (Bernardo et al., 2010). Hypertrophy can also be developed as a response to physiological stimuli such as chronic exercise. On the other hand, pathological hypertrophy is associated with an altered pattern of gene expression, presence of myofibrillar disarray, fibrosis, and contractile dysfunction and thus can lead to heart failure and sudden cardiac death (SCD) (Ho, 2010; Ho et al., 2010; Abel and Doenst, 2011). Most commonly hypertrophic cardiomyopathy (HCM) occurs in response to genetic mutations in all major sarcomeric proteins, including the myosin essential light chain (ELC) encoded by the *MYL3* gene (Alcalai et al., 2008).

There are number of studies demonstrating that physiological and pathological cardiac hypertrophy may be associated with distinct structural and functional as well as metabolic features (Abel and Doenst, 2011; Weeks and McMullen, 2011). It has also been shown that both physiologic and pathologic cardiac hypertrophy may display distinct biochemical and molecular signaling pathways (Iemitsu et al., 2001; Heineke and Molkentin, 2006; Bernardo et al., 2010). Pathological cardiac hypertrophy is manifested by alterations in cardiac contractile proteins [α-skeletal actin and the α- to β- myosin heavy chain (MHC) switch], increased expression of fetal genes such as atrial natriuretic peptide (ANP), B-type natriuretic peptide (BNP). The down- or up-regulation of calcium handling proteins such as the cardiac sarcoplasmic reticulum Ca^2+^-ATPase pump, SERCA2a can also be observed. Physiological cardiac hypertrophy may be associated with increased levels of peptide growth factors such as insulin-like growth factor-IGF 1 and epidermal growth factor and is often coupled to phosphatidylinositol 3-kinase (PI3K)/Akt pathway. It is known that many mutations in sarcomeric proteins cause cardiomyopathy and may ultimately initiate the hypertrophic gene remodeling program. Studies utilizing transgenic mouse models of hypertrophy are great tools for understanding the molecular pathways responsible for different forms of cardiac remodeling *in vivo*. Presented in this article study focuses on one particular sarcomeric protein, the essential light chain of myosin that is located in the neck region of the myosin cross-bridge (Rayment et al., 1993), and was shown to be important for myosin contractile function in health and in heart disease (Sawicki et al., 2005; Kazmierczak et al., 2009, 2013; Muthu et al., 2011; Cadete et al., 2012). Cardiac ventricular muscle exclusively expresses the long ELC isoform, containing the N-terminal extension, which was found to be important for the myosin-actin interaction during contraction and is expected to regulate heart performance (Winstanley et al., 1977; Sutoh, 1982; Henry et al., 1985; Trayer et al., 1987; Milligan et al., 1990; Morano et al., 1995; Timson et al., 1998; Morano, 1999; Timson, 2003). The structural modeling study by Morano (Aydt et al., 2007) depicts the N-terminus of the long myosin ELC as a rod-like 91 Å-long extension that can function as a bridge between the ELC core of the myosin head and the binding site of the ELC on the actin filament.

In this report we have studied cardiac remodeling in two forms of cardiac hypertrophy (pathological and non-pathological) related to the ventricular myosin ELC using transgenic mouse models carrying mutations in the human ventricular myosin ELC, Tg-A57G, and Tg-Δ43. The A57G (Alanine replaced by Glycine) variant of ELC was found in two unrelated Korean families and one Japanese patient diagnosed with HCM (review in Hernandez et al., 2007). The phenotype associated with this mutation was manifested as a classic asymmetric septal hypertrophy and SCD (Lee et al., 2001). In the Tg-Δ43 mouse model, the endogenous mouse ventricular ELC is partially replaced with the 43-amino-acid N-terminal truncated human ventricular ELC protein. We previously reported that Tg-Δ43 mice hypertrophied with age (>7 month-old), but the ventricles did not show any pathologic morphology. In support of the non-pathologic hypertrophy phenotype, an MRI (magnetic resonance imaging) assessment of Tg-Δ43 hearts demonstrated normal cardiac function compared to age matched controls (Kazmierczak et al., 2009).

Despite intensive research in many laboratories, questions about critical molecular mechanisms responsible for the transition from hypertrophy to heart failure still remain unanswered. Presented here transgenic mouse models carrying either the A57G mutation or the Δ43 truncation in ELC protein, generated in our laboratory (Kazmierczak et al., 2009, 2013; Muthu et al., 2011) represent different types of hypertrophy and therefore are valuable tools in understanding the pathological vs. non-pathological remodeling of the heart. We have examined the effects of both A57G and Δ43 mutations on heart remodeling before and after strenuous exercise and characterized the functional effects of these mutations in mice subjected to strenuous exercise by swimming. The observed effects were compared to those seen in Tg-WT (wild-type) mice expressing the full length non-mutated human ventricular ELC. We show that remodeling of the heart in Tg-A57G mice follows the pathological pathway leading to HCM, while cardiac phenotype observed in Tg-Δ43 mice is of non-pathological nature.

## Material and methods

### Transgenic mice

All animal studies were conducted in accordance with institutional guidelines and the protocol was reviewed and approved by the Animal Care and Use Committee at the University of Miami Miller School of Medicine (UMMSM). UMMSM has an Animal Welfare Assurance (A-3224-01, effective July 11, 2007) on file with the Office of Laboratory Animal Welfare (OLAW), National Institutes of Health. The generation and characterization of transgenic (Tg) mice used in this study and Tg protein expression profiles have been described earlier (Kazmierczak et al., 2009, 2013; Muthu et al., 2011). Previously produced Tg-WT lines, L1, L3, and L4 expressing 88, 30, and 77% of WT-ELC (UniProtKB: P08590) and Tg-A57G lines, L1, L2, and L5 expressing 80, 55, and 75% of A57G-mutant, respectively were used in this study. As for Tg-Δ43, two lines were used for this study, one previously generated expressing 40% of Δ43 protein (L9), and the second line expressing 55% transgene which was generated by cross breeding of existing mice (L8 × L8) in order to increase transgenic protein expression (Kazmierczak et al., 2009). The percent of protein expression indicates the amount of replacement of the endogenous mouse cardiac ELC by human ventricular WT (UniProtKB: P08590) or its two mutants. In all experiments Tg-A57G and Tg-Δ43 mice were gender and age matched with Tg-WT controls (Kazmierczak et al., 2009; Muthu et al., 2011; Kazmierczak et al., 2013).

Chronic 4 week-long training by swimming of Tg-WT, Tg-A57G, and Tg-Δ43 mice was performed in water at 30–32°C (to avoid thermal stress). Initial swim time was set as 10 min, thereafter gradually increasing until 90 min sessions were reached. The 90-min training schedule was continued twice a day (separated by 4–5 h), 7 days a week, for 4 weeks. This protocol was demonstrated to be highly effective in promoting physiological hypertrophy in mice (Evangelista et al., 2003; Galindo et al., 2009).

### Histological evaluation of the hearts from transgenic ELC mice

After euthanasia, the hearts from 3 to 5-month-old Tg-Δ43, Tg-A57G, and Tg-WT mice were excised, weighed and immersed in 10% buffered formalin. Slides of whole mouse hearts were prepared at the Histology Laboratory (University of Miami Miller School of Medicine, Miami FL). The paraffin-embedded longitudinal sections of left ventricles (LV) of H&E (hematoxylin and eosin) and Masson's trichrome stained hearts were examined for overall morphology and fibrosis using a Dialux 20 microscope, 40×/0.65 NA (numerical aperture) Leitz Wetzlar objective and an AxioCam HRc (Zeiss) as described previously (Kazmierczak et al., 2009, 2013; Muthu et al., 2011).

### Skinned papillary muscle strips from transgenic mice

The papillary muscles of the left ventricles from 3 to 5 months old Tg-A57G L1 mice, Tg-Δ43 (L9 and inbred cross of L8) and Tg-WT (L1, L3, and L4) mice were isolated, dissected into small muscle bundles and chemically skinned in 50% glycerol and 50% pCa 8 solution (10^−8^ M [Ca^2+^], 1 mM free [Mg^2+^] (total MgPr (propionate) = 3.88 mM), 7 mM EGTA, 2.5 mM [Mg-ATP^2−^], 20 mM Mops, pH 7.0, 15 mM creatine phosphate and 15 units/ml of phosphocreatine kinase, ionic strength = 150 mM adjusted with KPr) containing 1% Triton X-100 for 24 h at 4°C. Then the bundles were transferred to the same solution without Triton X-100 and stored at −20°C for 4–6 days (Kazmierczak et al., 2012).

### Steady-state force measurements

Small muscle strips ~1.4 mm in length and 100 μm in diameter were isolated from a batch of glycerinated skinned mouse papillary muscle bundles and attached by tweezer clips to the force transducer of the Guth Muscle Research System (Heidelberg, Germany). They were placed in a 1 ml cuvette and skinned in 1% Triton X-100 dissolved in pCa 8 buffer for 30 min, as described in Kazmierczak et al. (2012). Then they were rinsed in pCa 8 and their length adjusted to remove the slack. This procedure results in sarcomere length (SL) ~2.1 μm as judged by the first order optical diffraction pattern as described in Muthu et al. (2011); Wang et al. (2013a,b); Huang et al. (2014). Muscle strips were then tested for steady state force development in pCa 4 solution (composition is the same as pCa 8 buffer except the [Ca^2+^] = 10^−4^ M). All experiments were carried out at 21°C. Maximal steady state tension measured in pCa 4 solution was expressed in Newtons per cross section of the muscle strip (kN/m^2^). The measurement of diameter was taken at ~3 points along the muscle strip length with an SZ6045 Olympus microscope (zoom ratio of 6.3:1, up to 189x maximum magnification) and averaged (Muthu et al., 2012).

### The Ca^2+^ dependence of force development

After the initial steady state force was determined, muscle strips were relaxed in pCa 8 buffer and then exposed to solutions of increasing Ca^2+^ concentrations from pCa 8 to 4 (Dweck et al., 2005). Steady-state force was measured in each “pCa” solution followed by relaxation in pCa 8 solution. Data were analyzed using the Hill equation (Hill et al., 1980) where “[Ca^2+^]_50_ or pCa_50_” is the free Ca^2+^ concentration which produces 50% maximum force, and “n_H_” is the Hill coefficient.

### Assessment of myosin content in sedentary and exercised animals

Mouse cardiac myofibrils were prepared according to the method described previously (Kazmierczak et al., 2013). Briefly, after euthanasia, the hearts from 3 to 5 months old transgenic mice were isolated and frozen in liquid nitrogen and stored at −80°C until processed. For the preparation of myofibrils the tissue was thawed in the CMF (cardiac myofibril) buffer consisting 5 mM NaH_2_PO_4_, 5 mM Na_2_HPO_4_(pH 7.0), 0.1 mM NaCl, 5 mM MgCl_2_, 0.5 mM EGTA, 5 mM ATP, 5 nM microcystin, 0.1% Triton X-100, 20 mM NaF (phosphatase inhibitor), 5 mM DTT and 1 μl/ml protease inhibitor cocktail (Sigma-Aldrich Corp., St. Louis, MO, USA). The tissue was then homogenized in a Mixer-Mill MM301 (Retsch) until homogenous. The homogenate was centrifuged for 4 min at 8000 g and the supernatant was discarded. After centrifugation, the pellets were left on ice for 4 min. This step was repeated three times until the pellet turned white. The pellets were then resuspended in the CMF buffer and the myofibrils were subsequently dissolved in Laemmli sample buffer and loaded on 15% SDS-PAGE. The thick and thin filament proteins were detected by Coomassie brilliant blue staining. Band intensities were measured using Image J software and ratios of total myosin regulatory light chain (RLC_tot_) to tropomyosin (RLC_tot._/Tm) and RLC_tot._/TnI (troponin I) were determined.

### Myofilament protein phosphorylation in sedentary and exercised mice

Mouse cardiac myofibrils were used to determine sarcomeric protein phosphorylation. After separation of the samples on 15% SDS-PAGE Pro-Q Diamond phosphoprotein gel stain reagent (Invitrogen) was used (as described in the manufacturer's manual) to assess phosphorylation of troponin (TnT, TnI) and myosin RLC. The total protein was further detected in the same gel using the Coomassie brilliant blue staining. Myofilament protein phosphorylation ratio (ProQ) was calculated relative to the corresponding Coomassie brilliant blue staining (ProQ/Coomassie) using Image J software.

### Assessment of gene expression changes in the hearts of Tg-A57G and Tg-Δ43 mice

Total RNA was isolated from ventricles of sedentary and exercised Tg-WT, Tg-A57G, and Tg-Δ43 mice and converted to double stranded cDNAs using Random Primers and a High-Capacity cDNA Reverse Transcription Kit (Applied Biosystems) as described earlier (Kazmierczak et al., 2009). Quantitative PCR was conducted using SYBR Green I chemistry with gene-specific Quantitect Primer Sets (Qiagen) for murine: ANP (atrial natriuretic factor: NM_008725), BNP: NM_008726), Myh6 (α-myosin heavy chain, cardiac: NM_010856), Myh7 (β-MHC: NM_080728), ColVIIIa (collagen type 8a, NM_007739), and ATP2a2 (NM_001110140.3) which encodes two Ca^2+^-transporting ATPase isoforms, SERCA2a (cardiac) and SERCA2b (non-muscle). SERCA2a is the predominant Ca^2+^ pump in the myocardium (Vangheluwe et al., 2003). All reactions were performed in triplicate and run using BIO-RAD iQ5 Multicolor Real-Time PCR Detection System with the following cycle parameters: cycle of 50°C (2 min) followed by 95°C (10 min), 40 cycles of 95°C (15 s) followed by 60°C (1 min). Raw data were analyzed using the BIO-RAD CFX Manager Software, and fold change in expression of each gene was calculated using the relative quantification (RQ) ΔΔCt method with the levels of GAPDH (glyceraldehyde-3-phosphate dehydrogenase: NM_008084) as the normalizer gene (Kazmierczak et al., 2009).

### Statistical analysis

All data were expressed as mean ± s.e.m. Statistical comparisons were performed using ANOVA or independent *t*-test (Sigma Plot 11; Systat Software, San Jose, CA). *P*-values <0.05 indicated statistically significant differences.

## Results

### Histological evaluation of the hearts from Tg-A57G and Tg-Δ43 mice

**Figure 1:**
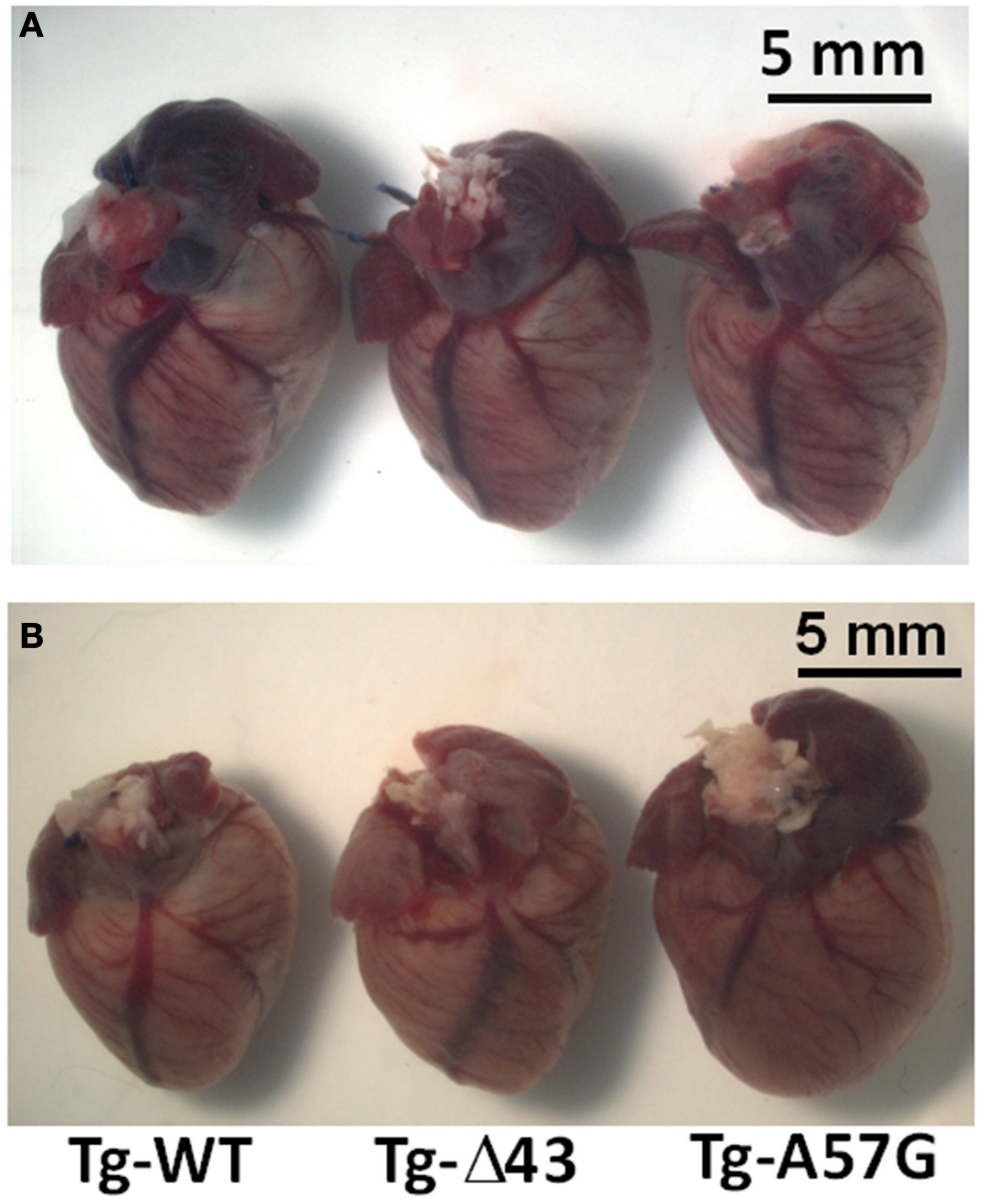
**Gross morphology of representative hearts from ~5 month-old sedentary (A) and ~3 month-old exercised (B) Tg-WT, Tg-Δ43 and Tg-A57G male mice**.

The evaluation performed for sedentary animals shows normal gross morphology for all tested 3–5 month-old male mice (Tg-WT, Tg-Δ43, and Tg-A57G) (Figure 1A). In our earlier study on sedentary Tg-Δ43 mice, we observed cardiac hypertrophy in mice ~7 months of age while the hearts of ~2 month-old Tg-Δ43 animals were indistinguishable from control Tg-WT mice (Kazmierczak et al., 2009). As we confirmed later, the hypertrophy in Tg-Δ43 mice appeared to be age dependent and profound hypertrophy was observed in ~12 month-old Tg-Δ43 mice compared with age matched Tg-WT hearts (Muthu et al., 2011). Thus, the lack of abnormal heart growth observed in 3–5 month-old sedentary Tg-Δ43 mice (Figure 1A), is not surprising and suggests that Tg-Δ43 mice have to be at least 7 months of age to develop cardiac hypertrophy. Regarding sedentary Tg-A57G mice, similar to what we reported earlier (Muthu et al., 2011), no cardiac growth could be observed in 3–5 month-old mice (Figure 1A) or the mice as old as ~12 months of age (Muthu et al., 2011). However, exercised 3–5 month-old Tg-A57G animals do show cardiac hypertrophy while the hearts of Tg-Δ43 mice were comparable in size to Tg-WT controls (Figure 1B).

**Figure 2:**
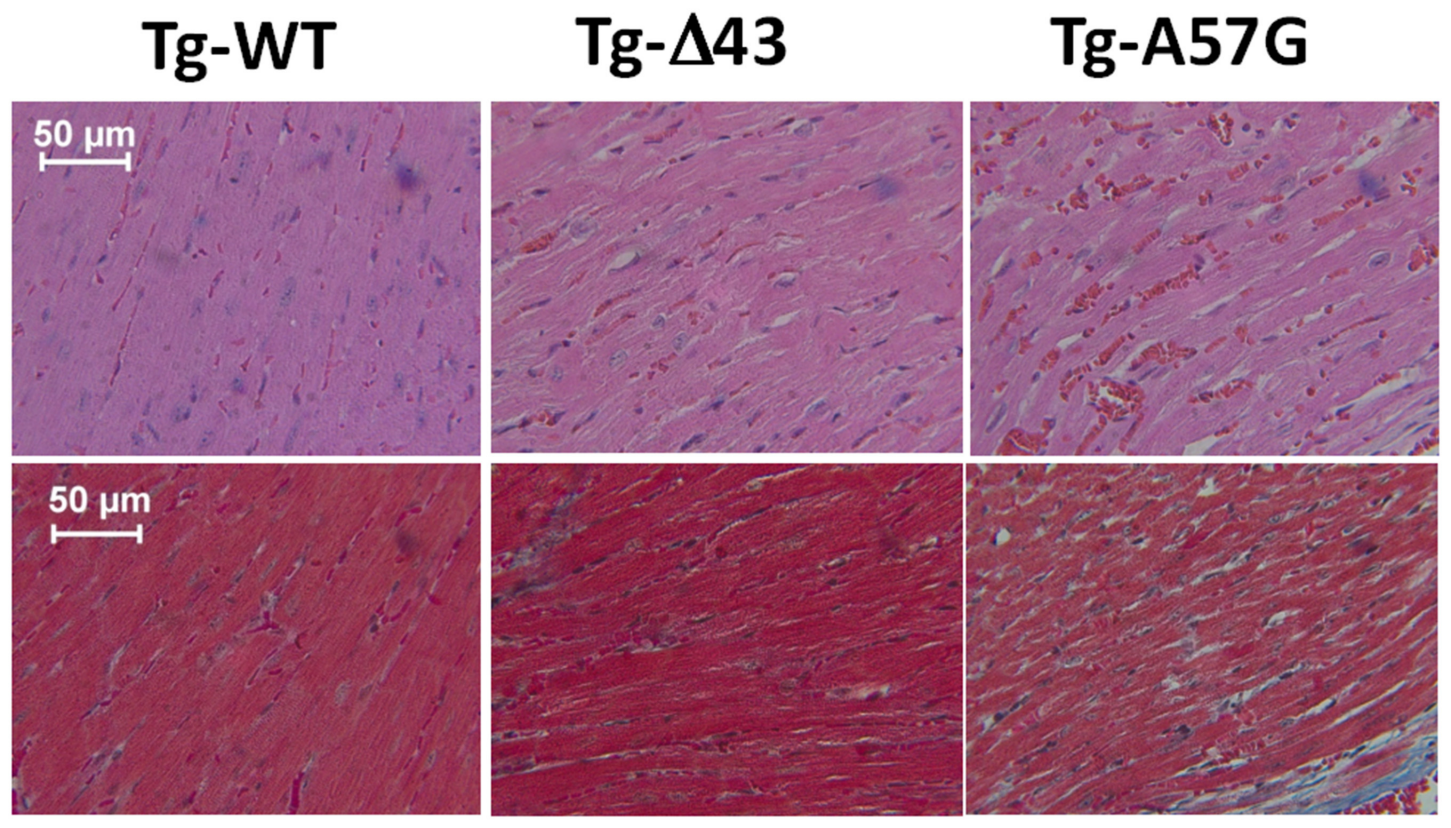
**H&E (upper panel) and Masson's trichrome (bottom panel) stained LV sections from exercised ~3 month-old Tg-WT, Tg-Δ43, and Tg-A57G male mice**.

Histology examination of animals subjected to strenuous exercise is presented in Figure 2. H&E and Masson's trichrome stained left ventricular (LV) sections from Tg-Δ43 hearts revealed that the hearts of exercised 3–5 month-old mice demonstrated no tissue abnormalities, myofilament disarray or fibrosis compared to Tg-WT controls. In contrast, the evaluation of LV heart sections from Tg-A57G mice showed occurrences of fibrosis, especially in the interventricular septum compartment (Figure 2). This is in accord to previously reported pathological morphology in sedentary Tg-A57G mice, which included extensive disorganization of myocytes and interstitial fibrosis (Muthu et al., 2011; Kazmierczak et al., 2013).

### Signaling pathways and changes in the gene expression profile in the hearts of two different models of hypertrophy

**Figure 3:**
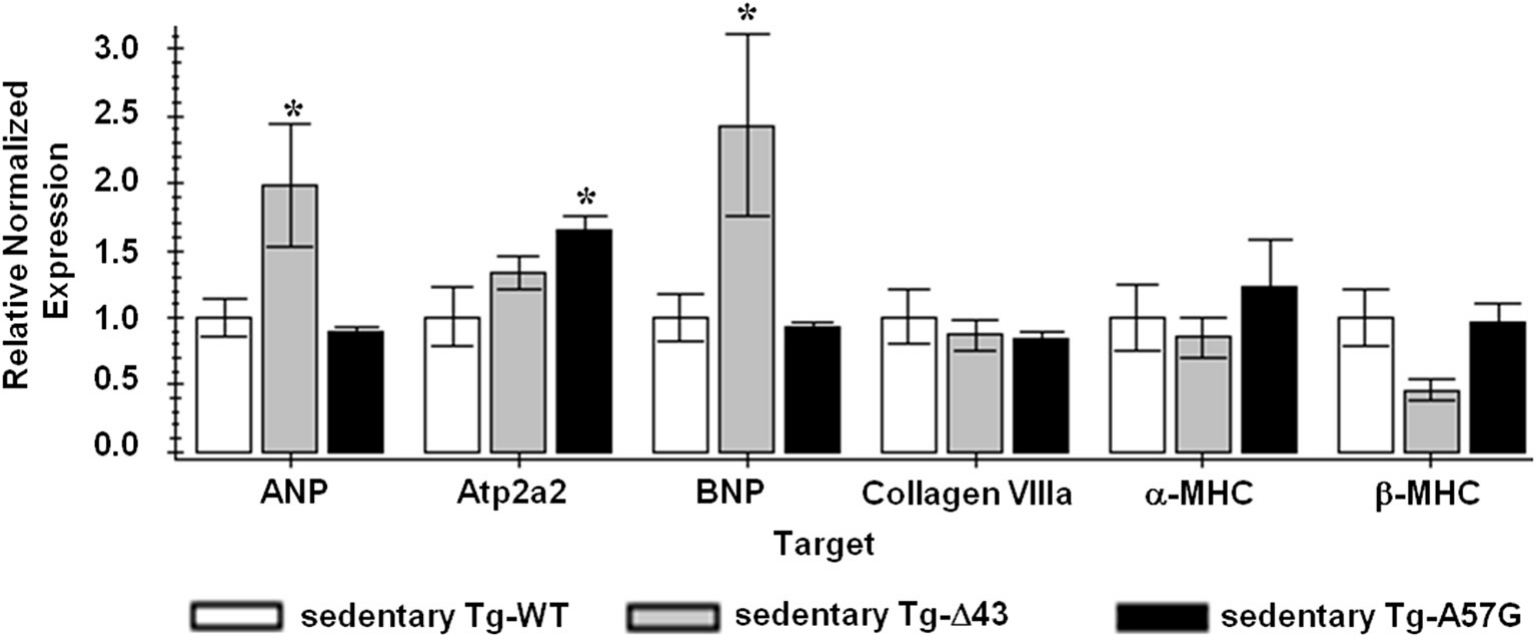
**QPCR data for sedentary ELC animals**. Sedentary Tg-WT was used as a control sample (FC = 1); ^*^indicates *P* < 0.05 compared to sedentary Tg-WT.

**Figure 4:**
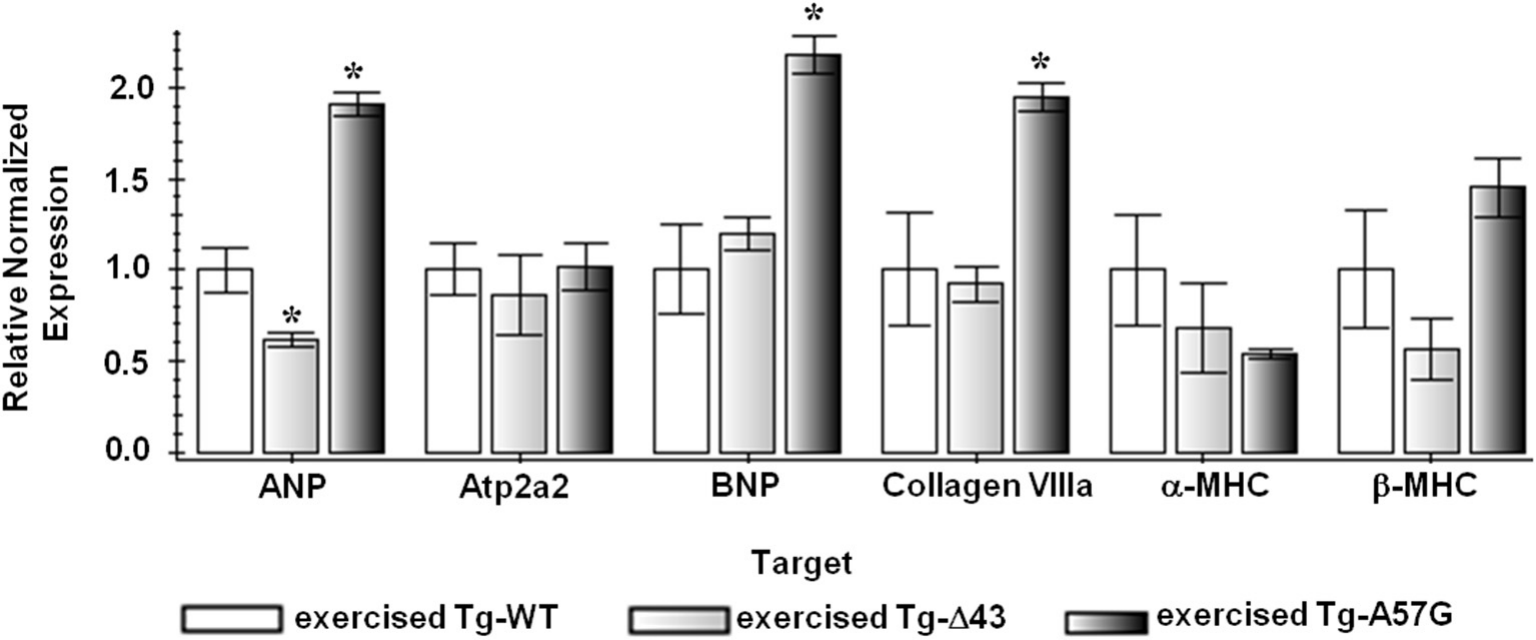
**QPCR data for exercised ELC animals**. Exercised Tg-WT is used as a control sample (FC = 1); ^*^indicates *P* < 0.05 compared to exercised Tg-WT mice.

**Figure 5:**
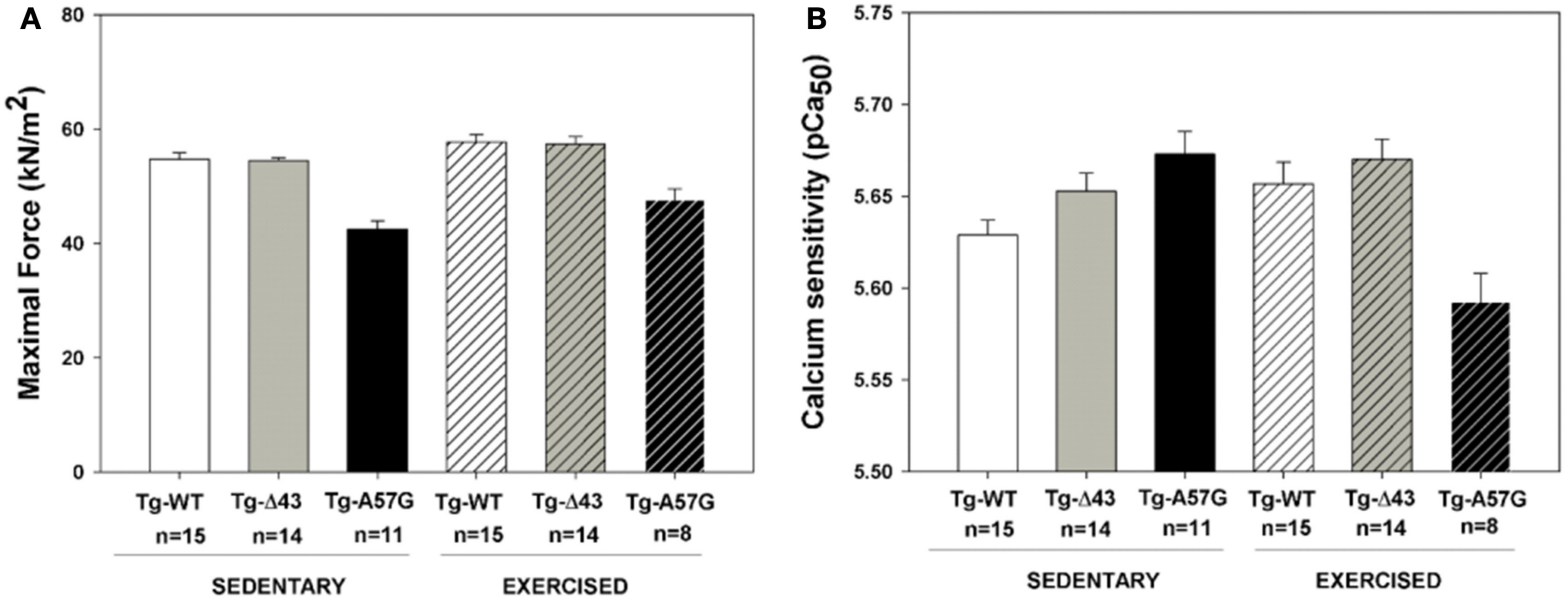
**Measurements of steady-state force in skinned papillary muscle strips from sedentary and exercised transgenic ELC male mice. (A)** Maximal force assessment at pCa4. **(B)** Calcium sensitivity of force.

Hypertrophic remodeling in Tg-Δ43 and Tg-A57G animal models was studied by looking at mutation-induced alterations in cardiac contractile proteins (α- to β-MHC switch), expression of the fetal genes such as ANP and BNP. We also examined whether cardiac remodeling in these mice involved changes in the calcium handling proteins such as SERCA2a. Studies of gene expression using real-time PCR in mouse myocardium were performed for sedentary and exercised Tg animals and the results are presented in Figures 3, 4, respectively. For sedentary Tg-A57G mice, the expression of sarcoplasmic ATP2a2 was higher compared with sedentary Tg-WT (~1.6 fold). The upregulation of mRNA SERCA2a in Tg-A57G mice (Figure 3) may translate to a faster relaxation of the A57G myosin cross-bridges and potentially lower contractile force generation in Tg-A57G mice, what is actually observed (Figure 5A). For sedentary Tg-Δ43 mice the expression levels of ANP and BNP were higher compared with sedentary Tg-WT (2- and 2.4 fold, respectively) (Figure 3).

This upregulation of both natriuretic peptides in Tg-Δ43 mice most likely manifests their protective role against excessive cardiac remodeling and preventing uncontrolled myocardial growth of Tg-Δ43 hearts (Tsuruda et al., 2002; Gardner, 2003).

To evaluate a combined effect of strenuous exercise and ELC mutation on heart remodeling we have also studied the molecular mechanisms and signaling pathways that change due to exercise. Figure 4 presents the gene expression analysis using real-time PCR in mouse myocardium performed for exercised Tg ELC animals. In Tg-A57G mice the exercise training reactivated the fetal gene program and upregulation in the β-MHC, ANP and BNP was observed. Expression of ANP and BNP was respectively 1.9- and 2.1 fold higher compared with exercised Tg-WT (both differences statistically significant *P* < 0.05). Additionally, upregulation of collagen VIIIa (1.9 fold change, significant *P* < 0.05) and β-MHC (not significant) and downregulation of α-MHC was observed for exercised Tg-A57G compared with exercised Tg-WT mice (Figure 4). On the other hand, exercised Tg-Δ43 mice showed a 0.6-fold lower expression level of ANP (*P* < 0.05) and no change in BNP marker compared to exercised Tg-WT controls (Figure 4). Changes in gene expression observed in Tg-A57G mice in response to strenuous exercise represent a pathologic type of remodeling occurring in the disease mouse model. In contrast, lack of changes in expression levels of BNP, β-MHC and collagen VIIIa in exercised Tg-Δ43 mice represents an adaptive response to exercise and non-pathological remodeling in Tg-Δ43 animals.

### Steady-state force measurements and Ca^2+^ dependence of force development

**Figure 6:**
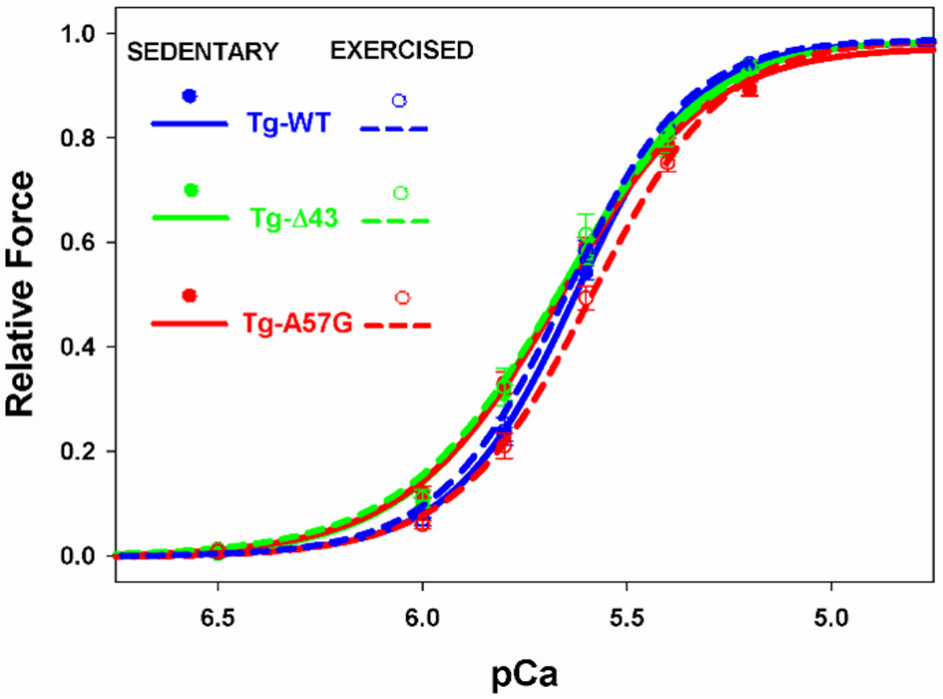
**The force- pCa relationship in transgenic skinned mouse papillary muscle strips from Tg-WT, Tg-Δ43, and Tg-A57G male mice**.

**Figure 7:**
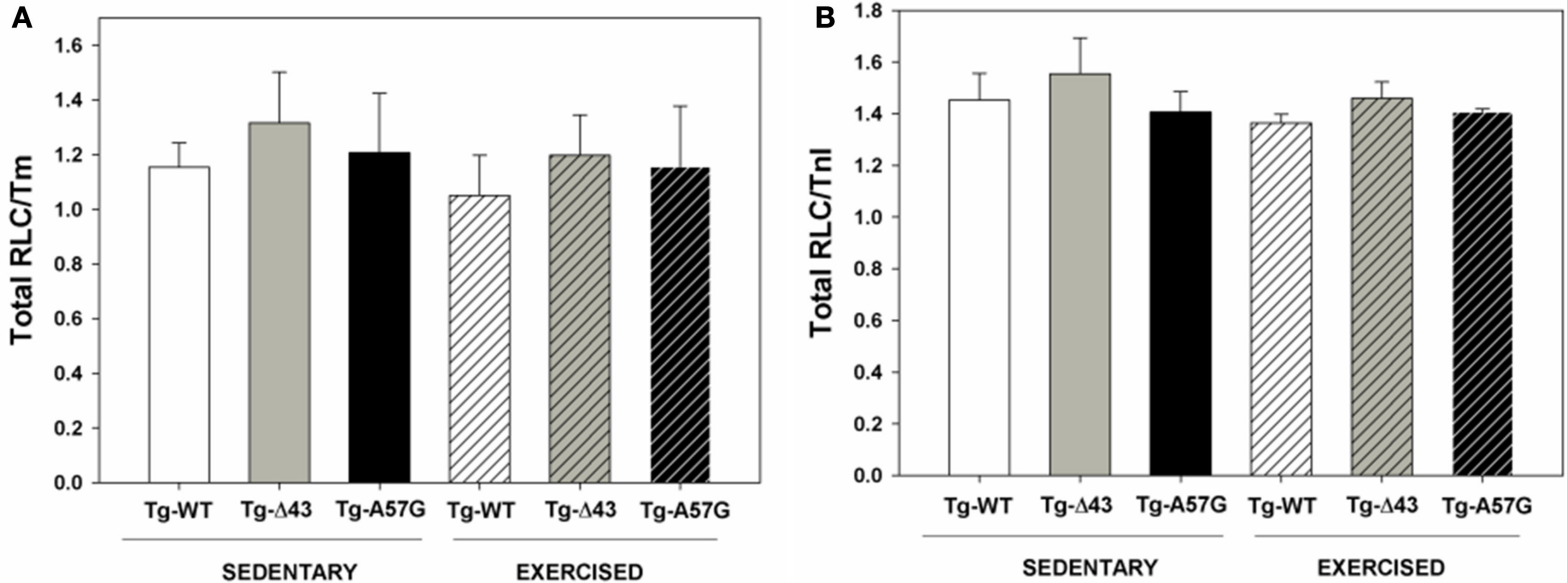
**Thick and thin filament protein content in sedentary and exercised Tg-Δ43 and Tg-A57G mice compared to control Tg-WT**.

The data from functional measurements assessed in skinned mouse papillary muscle strips from Tg-Δ43 and Tg-A57G animals are presented in Figure 5, and the values of maximal force and pCa_50_ are summarrized in Table 1. Different pattern of contractile force generation and the Ca^2+^ sensitivity of force was observed betweenTg-A57G and Tg-Δ43 mice. Similar to our earlier study (Kazmierczak et al., 2013), sedentary Tg-A57G mice showed the lowest maximal force (~20% lower vs. sedentary Tg-WT) and a slightly increased Ca^2+^ sensitivity of force (ΔpCa_50_ ≅0.04 vs. sedentary Tg-WT), both changes statistically significant. As we demonstrated earlier and in this study, sedentary Tg-A57G animals showed reduced maximal (pCa 4) force and increased Ca^2+^ sensitivity following the pattern of pathological hypertrophy (Muthu et al., 2011; Kazmierczak et al., 2013) (Figures 5, 6). Contrary, maximal force for sedentary Tg-Δ43 mice was similar to that of Tg-WT and the Ca^2+^ sensitivity was not changed (ΔpCa_50_ ≅0.02; not statistically significant) compared to Tg-WT controls. The previously reported reduction in steady-state force in ~2 month-old and ~7 month-old Tg-Δ43 mice (Kazmierczak et al., 2009) was likely associated with a decrease in the myosin content observed in these animals vs. the lack of changes in myosin expression seen in the current investigation (Figure 7).

**Table 1:** **Maximal pCa 4 force and calcium sensitivity of force in transgenic ELC skinned mouse papillary muscle strips**.

**System**	**pCa_50_**	**F_max_ (kN/m^2^)**
Sedentary Tg-WT	5.629 ± 0.008	54.804 ± 1.133
Sedentary Tg-Δ43	5.653 ± 0.010	54.581 ± 0.399
Sedentary Tg-A57G	5.673 ± 0.012	42.482 ± 1.458
Exercised Tg-WT	5.657 ± 0.012	57.731 ± 1.273
Exercised Tg-Δ43	5.670 ± 0.011	57.421 ± 1.279
Exercised Tg-A57G	5.592 ± 0.016	47.615 ± 1.967

Data are mean ± SEM of n = 8–15 individual strips for each group of mice.

In response to exercise, all transgenic animals showed a higher level of maximal force per cross-section of muscle strip vs. their sedentary controls (Table 1). Similarly to the direction of changes observed for sedentary animals, in response to swimming, the maximal force for Tg-Δ43 was similar while that for Tg-A57G was significantly reduced (~20% lower, *P* < 0.005) compared to exercised Tg-WT. Additionally, for exercised Tg-A57G, the Ca^2+^ sensitivity of force was significantly lower compared to sedentary Tg-A57G mice (ΔpCa_50_ ≅0.08) (Figures 5B, 6 and Table 1). Tendency toward desensitization of myofilaments to Ca^2+^ observed in Tg-A57G mice (Table 1) may represent a key step in the signaling pathway for the pathological hypertrophic remodeling in this animal model of HCM. As expected, there were no significant changes in the Ca^2+^ sensitivity of force between exercised and sedentary Tg-WT mice. Likewise, no significant changes in the Ca^2+^ sensitivity were present between the exercised and sedentary Tg-Δ43 mice (Table 1). No changes in the cooperativity (Hill coefficient) values for all groups of mice (n_H_ = 2.6 ± 0.3) were noted (Figure 6).

### Assessment of myosin content in sedentary and exercised animals

As mentioned above, the reduced level of maximal contractile force measured in our previous study for sedentary Tg-Δ43 mice was most likely associated with deficiency in myosin content detected in the myocardium of Tg-Δ43 vs. Tg-WT mice (Kazmierczak et al., 2009). Therefore, myosin content in sedentary and exercised animals was assessed in this study and correlated with the ability of the myocardium to develop contractile force. Figure 7 presents the ratios of RLC_tot._/Tm and RLC_tot._/TnI determined in cardiac myofibrillar preparations from sedentary and exercised Tg-WT, Tg-A57G, and Tg-Δ43 mice. Compared to respective Tg-WT myofibrils (100%), the ratios of RLC_tot._/Tm and RLC_tot._/TnI obtained for sedentary and exercised Tg-A57G mice were similar to Tg-WT indicating no change in myosin expression in all tested animals (Figure 7). Therefore, the lower maximal force observed for the A57G mutation in this study is not due to the lower myosin content but due to defective function of the A57G-myocardium. The values of RLC_tot._/Tm and RLC_tot._/TnI ratios obtained for sedentary and exercised Tg-Δ43 mice were also very similar to Tg-WT (~110%), indicating no previously observed deficiency of myosin in the hearts of Tg-Δ43 animals (Kazmierczak et al., 2009).

### Myofilament protein phosphorylation in sedentary and exercised mice

**Figure 8:**
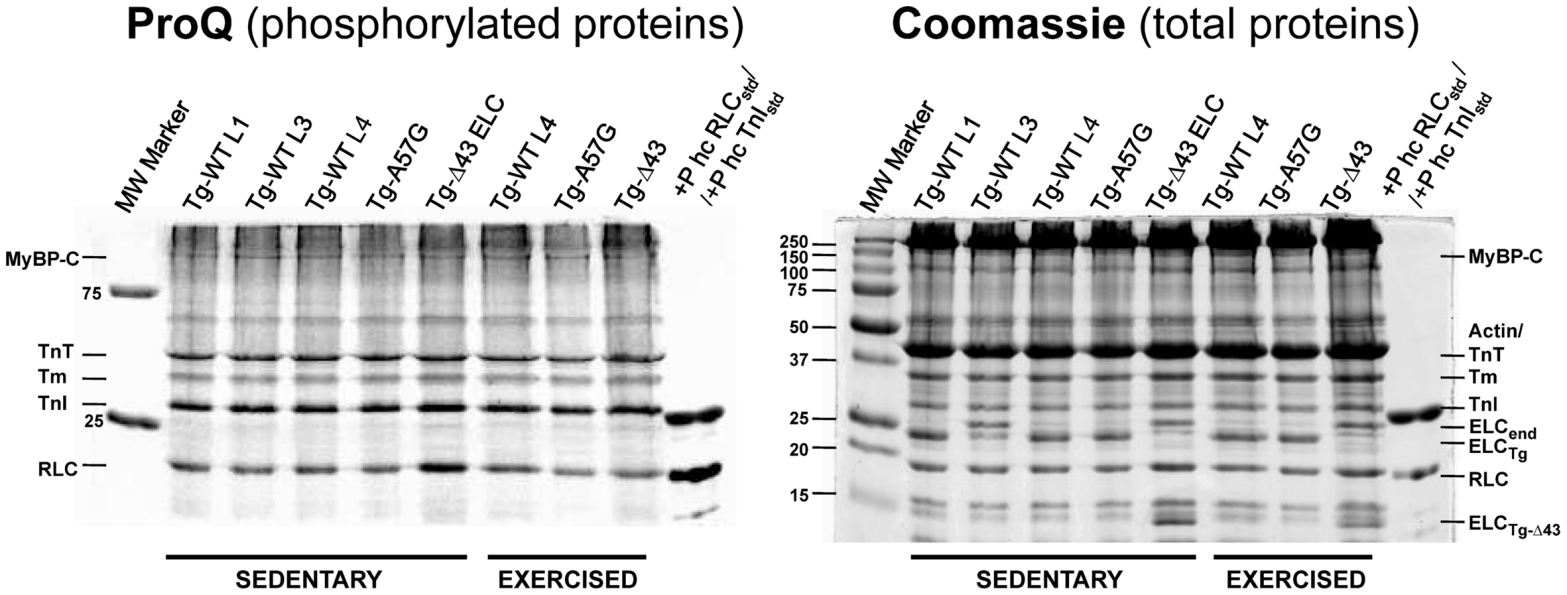
**Assessment of protein phosphorylation in sedentary and exercised Tg-WT, Tg-Δ43, and Tg-A57G mouse myofibrils**. MyBP-C, myosin binding protein C; TnT, troponin T; Tm, tropomyosin; TnI, troponin I; RLC, myosin regulatory light chain; ELC_end_, myosin ELC (all mouse isoforms); ELC_Tg_, transgenic human ELC; +P hc std, phosphorylated human cardiac protein standard.

The force-generating capacity of sarcomeres has been known to be tightly regulated by sarcomeric proteins phosphorylation (Sweeney et al., 1993; Sadayappan et al., 2009; Nixon et al., 2012). To test whether the ELC mutation (Δ43 or A57G) and/or exercise had any effect on phosphorylation of sarcomeric proteins, we have examined phosphorylation of myosin binding protein C (MyBP-C), Tm, troponin (TnT and TnI) and myosin RLC in mouse cardiac myofibrils using the Pro-Q Diamond staining. As shown in Figure 8, no significant changes in endogenous MyBP-C, Tm, TnT, TnI, or RLC phosphorylation were found in sedentary or exercised Tg-Δ43 or Tg-A57G animals compared to Tg-WT mice. The lack of the effect of exercise on protein phosphorylation is somewhat surprising but has been observed by other laboratories. No effect of exercise on phosphorylation of myosin RLC in the ventricles or atria of mice was found by Fewell et al. (1997). Likewise, no changes in phosphorylation of titin and TnI in mice upon exercise training were recently reported by the Granzier group (Hidalgo et al., 2014). These results suggest no immediate molecular contacts between the ELC protein and the phosphorylation domains of MyBP-C, Tm, TnT, TnI, or RLC.

## Discussion

Hypertrophic remodeling is a highly important phenomenon; however, the molecular mechanisms and signaling pathways underlying these processes have not yet been fully understood. In particular, molecular pathways that play a causal role in the development of pathological vs. physiological hypertrophy need to be recognized due to the known association between cardiac hypertrophy and nearly all forms of heart failure (Levy et al., 1990). Recognition of functional, structural, metabolic, and molecular differences between pathological and non-pathological hypertrophy may help to develop potential therapeutic approaches to benefit patients with HCM and heart failure.

### Remodeling in sedentary mice

**Figure 9:**
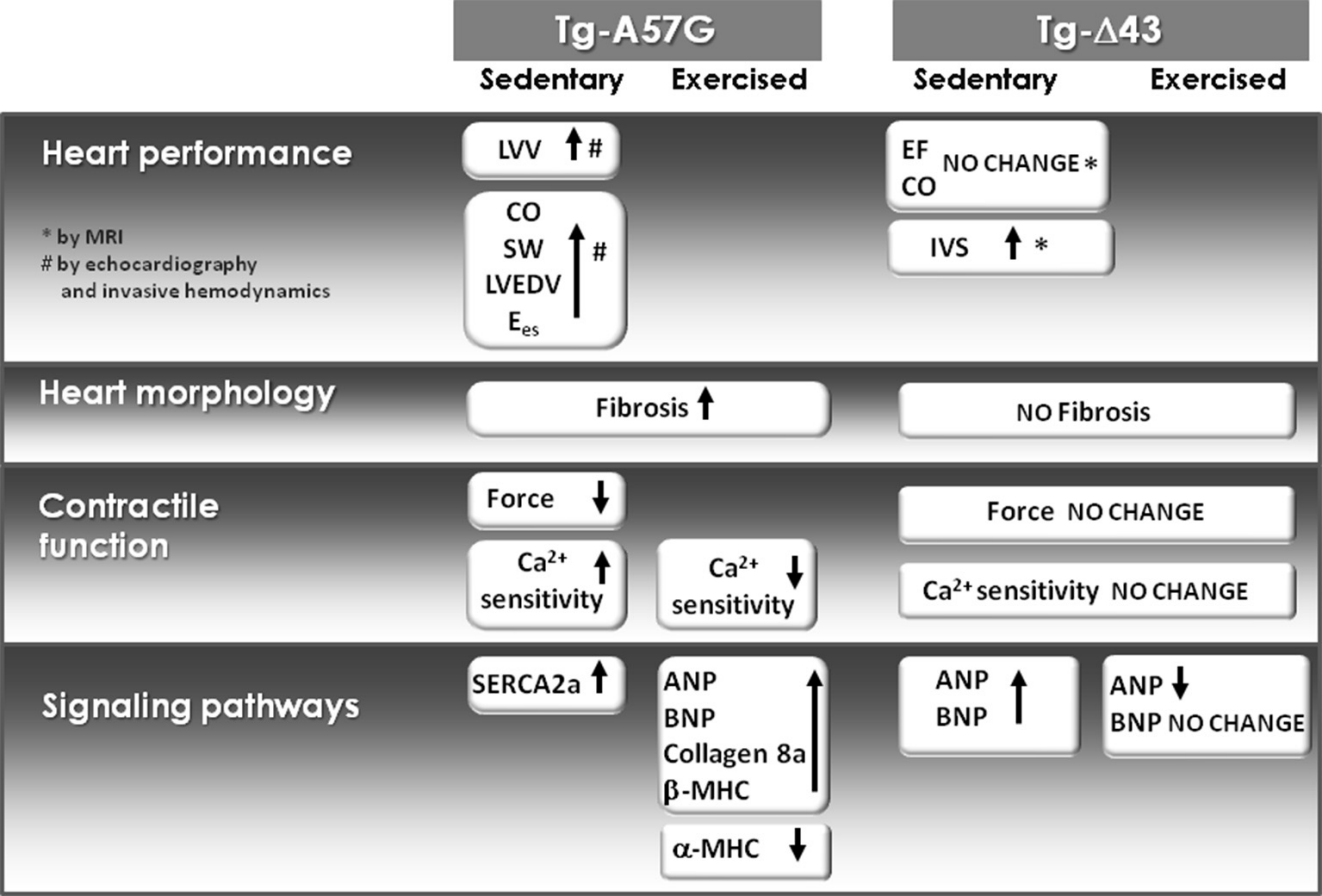
**Molecular effects of A57G and Δ43 mutations in the myosin ELC on heart morphology and function and expression of hypertrophic markers in sedentary and exercised Tg ELC mice**. All data are compared with either sedentary or exercised Tg-WT ELC. The *in vivo* data were published previously. LVV, LV-volume; CO, cardiac output; SW, stroke work; LVEDV, LV-end diastolic volume; E_es_, measure of myocardial contractility defined by the slope of the end-systolic pressure-volume relationship.

Since the ELC is a highly essential element of the myosin molecule, its structure and the interaction with the MHC and actin play important roles in the sarcomeric assembly and force production (Hernandez et al., 2007). This investigation was designed to look at two different cardiac phenotypes induced by structural alterations in the myosin ELC in mouse models of pathologic (Tg-A57G) and physiologic (Tg-Δ43) HCM. The effects of both ELC mutations on heart function and cardiac remodeling are summarized in Figure 9. Data obtained for sedentary and exercised Tg-A57G and Tg-Δ43 animals show clear differences between these two phenotypes. Consistent with our earlier reports, we monitored significantly compromised contractile function (lower maximal force and higher Ca^2+^ sensitivity) in sedentary Tg-A57G mice, which coincided with upregulation of the calcium handling protein SERCA2a compared to sedentary Tg-WT animals. It has been reported that in animal models, severe compensated hypertrophy in response to pressure overload is accompanied by a large decrease in the mRNA and protein content of SERCA2a (Buttrick et al., 1994; Rockman et al., 1994). However, in rats with moderate cardiac hypertrophy, the level of SERCA2a mRNA or protein was unaltered or even upregulated (Rockman et al., 1994; Arai et al., 1996). SERCA2a is a key protein responsible for maintaining a balanced concentration of Ca^2+^ during the cardiac cycle and it controls the transport of Ca^2+^ to the SR during relaxation. The upregulation of mRNA SERCA2a in Tg-A57G mice (Figure 3) may translate to an increased protein activity, which would lead to faster relaxation of the A57G myosin cross-bridges and potentially lower contractile force generation in Tg-A57G myocardium (Figure 5A).

These adverse effects were not present in sedentary Tg-Δ43 mice, the model of non-pathological cardiac phenotype. The maximal force and Ca^2+^ sensitivity values were similar to those determined for sedentary Tg-WT mice, indicating a normal heart function in Tg-Δ43 animals. As we reported earlier (Kazmierczak et al., 2009, 2013; Muthu et al., 2011) both mouse models demonstrated cardiac hypertrophic growth, but the pathological features were only observed in sedentary A57G animals (Kazmierczak et al., 2009; Muthu et al., 2011; Kazmierczak et al., 2013). Discrepancies exist regarding the lack of reduction in maximal force observed in Tg-Δ43 mice in the current study (Table 1) and the previously reported decrease in steady-state force in Tg-Δ43 mice (Kazmierczak et al., 2009). The reason for a lower force reported in Kazmierczak et al. (2009) was an approximately 30% deficit in myosin cross-bridge content in the myocardium of Tg-Δ43 mice (Kazmierczak et al., 2009). This deficit in myosin content was not observed in the current work (Figure 7), indicating a compensatory response in transgenic Tg-Δ43 mice over time. Consequently, the lack of abnormalities in myosin expression was most likely responsible for no changes in force per cross-section of muscle seen in Tg-Δ43 mice in the current investigation (Table 1). Additionaly, recently published data utilizing the same Tg-Δ43 mouse model, showed only a slight (and not significant) decrease in maximal tension between Tg-WT and Tg-Δ43 (Michael et al., 2013; Wang et al., 2013a).

Sedentary Tg-Δ43 mice also showed an upregulation of ANP and BNP (Figure 3). Upregulation of these transcripts is considered a highly conserved feature of ventricular hypertrophy (Lee et al., 1988; Dagnino et al., 1992; Buttrick et al., 1994; Nakagawa et al., 1995), but there are contradictory reports in the literature on the correlation of upregulation of natriuretic peptides with physiological hypertrophy that can be induced by various training programs in animals. Some publications report an upregulation (Buttrick et al., 1994; Allen et al., 2001), while some report no change (Azizi et al., 1995; Jin et al., 2000) or downregulation (Diffee et al., 2003) of ANP and/or BNP expression in these animal models. Both natriuretic peptides were shown to function as modulators of cardiac hypertrophy preventing uncontrolled myocardial growth and regulating ventricular remodeling secondary to compensatory hypertrophy (Tsuruda et al., 2002; Gardner, 2003). It is therefore possible that in the case of sedentary Tg-Δ43 mice, mild upregulation of ANP and BNP manifests their protective role against excessive cardiac remodeling and controlling the extent of cardiac hypertrophy.

There are some discrepancies between our earlier study and current investigation on the hypertrophic growth in sedentary Tg-Δ43 mice. In Kazmierczak et al. (2009), we presented the gross morphology images of young (~2 mo-old) and adult (~7 mo-old) Tg-Δ43 animals. The hypertrophy was only noted for the older animals. The hearts of young Tg-Δ43 mice were indistinguishable from age matched control Tg-WT mice. In the current study, the hearts of 3–5 month-old sedentary Tg-Δ43 mice were not much different than those of age matched Tg-WT controls (Figure 1A). These data suggest that hypertrophy in Tg-Δ43 mice is an age dependent process and the fact that it was not present in <5 month-old Tg-Δ43 animals (Figure 1A), i.e., ~2 months before the apparent threshold when the animals reach ~7 months of age (Kazmierczak et al., 2009) is not surprising. In line with this, a profound hypertrophy in ~12 month-old Tg-Δ43 mice was recently reported by our group (Muthu et al., 2011).

### Remodeling in exercised mice

Swimming exercise is known for its efficiency in inducing cardiac hypertrophy and significant increases in LV end-diastolic volumes in rats (Geenen et al., 1988). We hypothesized that strenuous exercise applied to Tg-Δ43 and Tg-A57G mice would lead to differential cardiac remodeling in these animal models displaying two distinct phenotypes of a “healthy” (Δ43) vs. “diseased” (A57G) heart. Our data on exercised animals confirmed our hypothesis. For Tg-A57G animals, we observed a significant upregulation of hypertrophic markers that were activated upon exercise initiating pathological hypertrophic remodeling in Tg-A57G mice (Figure 9). Upregulation of ANP and BNP transcripts observed in exercised Tg-A57G mice (Figure 4) manifests their sensitivity in monitoring ventricular hypertrophy (Lee et al., 1988; Dagnino et al., 1992; Nakagawa et al., 1995). ANP and BNP are released by the heart usually in response to heart failure (cardiac injury). The BNP levels in plasma have been shown to be useful in monitoring therapy for heart failure and typically a good response to effective treatment correlates with decreased concentrations of BNP (Lee et al., 2002; Yoshimura et al., 2002). Upregulation of ANP, BNP, and Collagen VIIIa was paralleled by a visible increase in heart size in Tg-A57G mice (Figure 1B), manifesting pathological hypertrophic remodeling in Tg-A57G mice (Figure 9).

Force generation and the Ca^2+^ sensitivity of force in Tg-A57G mice were significantly reduced after strenuous excise (Tables 1, 2) manifesting pathological hypertrophic remodeling. Although maximal force in these animals was slightly improved compared to sedentary mice it was still significantly lower than that monitored for other exercised mice. It is possible that strenuous exercise caused activation of the β-adrenergic pathway in Tg-A57G hearts that could lead to compromised heart performance. Decreased Ca^2+^ sensitivity in these animals compared to sedentary Tg-A57G mice might be indicative of progressing toward heart failure as observed in animal models of HF (Cazorla et al., 2005; Belin et al., 2007) and in humans (Van Dijk et al., 2009; Hoskins et al., 2010; Sequeira et al., 2013; Witjas-Paalberends et al., 2013). The presence of collagen deposits in the myocardium of Tg-A57G mice (Figure 2) indicates the propensity of the heart to increase its LV stiffness that ultimately may result in diastolic dysfunction in Tg-A57G mice (Wu et al., 2007).

**Table 2:** **Statistics for measurements of steady-state force in skinned papillary muscle strips from sedentary and exercised transgenic ELC animals**.

**Statistics (*P*-value)**			
	**Tg-WT ELC**	**Tg-Δ43**	**Tg-A57G**
**pCa_50_**			
Sedentary WT vs. mutant	N/A	0.0676	0.0049
Exercised WT vs. mutant	N/A	0.4212	0.0037
Sedentary vs. exercised	0.0601	0.2488	0.0008
**F max**			
Sedentary WT vs. mutant	N/A	0.8580	<0.0001
Exercised WT vs. mutant	N/A	0.8648	0.0002
Sedentary vs. exercised	0.0970	0.0437	0.0466

A different response on the strenuous exercise was observed for the second Tg-Δ43 animal model. Function of the heart remained normal and both maximal force and the Ca^2+^ sensitivity were similar to exercised Tg-WT mice. Additionally, exercised Tg-Δ43 animals showed similar pattern of gene expression to that observed in exercised Tg-WT mice, indicating that hypertrophic remodeling of the heart was no longer present in this animal model. The lack of changes in expression levels of BNP, β-MHC, and collagen VIIIa in exercised Tg-Δ43 mice represents an adaptive response to exercise and non-pathological remodeling in Tg-Δ43 animals compared to detrimental heart remodeling observed in exercised Tg-A57G mice.

## Conclusive remarks

Our previous X-ray studies on sedentary Tg-A57G and Tg-Δ43 mice demonstrated a mutation-induced decrease in the filament lattice spacing in skinned papillary muscles of Tg-A57G compared to Tg-WT controls (Muthu et al., 2011). These structural changes may initiate hypertrophic remodeling of the heart triggered by mutation-induced conformational changes within the sarcomere that lead to abnormal interaction of two major contractile proteins, myosin and actin causing abnormalities in contractile force production and heart performance. Pathological ventricular remodeling and anatomical changes observed in Tg-A57G hearts mostly develop in older animals and, as we anticipated, they were augmented in response to strenuous exercise. In contrast, X-ray diffraction studies on Tg-Δ43 mice demonstrated a shift of cross-bridge mass from the thick filament backbone toward the thin filaments and no changes in the interfilament spacing compared with Tg-WT controls (Muthu et al., 2011). These data indicate that the lack of the N-terminal ELC extension in Tg-Δ43 animals may lead to changes in myosin head orientation positioning it closer (at smaller angle) to the actin filaments (Muthu et al., 2011). The important function of the N-terminal extension of the ELC protein was also demonstrated in other study showing that the length-dependent activation in Tg-Δ43 strips was blunted in Tg-Δ43 animals (Michael et al., 2013). This could eventually lead to hypertrophy in Tg-Δ43 animals but of the non-pathological nature.

Combined knowledge of the genetic basis of HCM with biophysical and transcriptional analyses may help to better understand the pathophysiology of heart remodeling and make predictive correlations between the specific mutations with disease prognosis. The heart may tolerate various types of stimuli, but compensatory/adaptive responses that aim to maintain function may fail in case of patients with pre-existing HCM (hearts with disease-causing mutations), resulting in a wide range of functional deficits and/or cardiomyopathy. The strenuous exercise of competitive athletes has been known to make symptoms of HCM more apparent and because of the risk of SCD during exercise, HCM patients are excluded from most competitive sports (Michels et al., 2009). Our data confirm that it is reasonable to caution people with diagnosed genetic HCM against intensive exercise programs. Our previous and current studies suggest that the A57G mutation can initiate pathologic cardiac remodeling and drive the heart toward cardiomyopathy disease and/or heart failure while the Δ43–dependent pathway displays a tendency toward a non-pathological cardiac phenotype.

## Author contributions

Katarzyna Kazmierczak, Conception and design of research, execution of experiments, analysis and interpretation of data, figure preparation, drafting of the manuscript. Chen-ching Yuan, Execution of experiments, analysis of data. Jingsheng Liang, Execution of experiments, analysis of data. Wenrui Huang, Execution of experiments, analysis of data, figure preparation. Ana I. Rojas, Breeding mouse colonies and *in vivo* data collection. Danuta Szczesna-Cordary, Conception and design of research, analysis and interpretation of data, figure preparation, drafting of the manuscript.

## Funding

This work was supported by the National Institutes of Health Grants R01 HL-108343 and HL-071778 (to DSC).

### Conflict of interest statement

The authors declare that the research was conducted in the absence of any commercial or financial relationships that could be construed as a potential conflict of interest.
